# Male mice retain a metabolic memory of improved glucose tolerance induced during adult onset, short-term dietary restriction

**DOI:** 10.1186/2046-2395-1-3

**Published:** 2012-09-03

**Authors:** Kerry M Cameron, Satomi Miwa, Cornelia Walker, Thomas von Zglinicki

**Affiliations:** 1 Ageing Research Laboratories, Centre for Integrated Systems Biology of Ageing and Nutrition, Institute for Ageing and Health, Newcastle University, Newcastle upon Tyne, NE4 5PL, UK

**Keywords:** Dietary restriction, Glucose tolerance, Insulin sensitivity, Crossover, Metabolic memory, Sexual dimorphism, Body mass, Hyperphagia, Mouse

## Abstract

**Background:**

Chronic dietary restriction (DR) has been shown to have beneficial effects on glucose homeostasis and insulin sensitivity. These factors show rapid and robust improvements when rodents were crossed over from an *ad libitum* (AL) diet to DR in mid life. We aimed to determine whether the beneficial effects induced by short-term exposure to DR can be retained as a ‘metabolic memory’ when AL feeding is resumed (AL-DR-AL) and vice versa: whether the effects of long-term DR can be reversed by a period of AL feeding (DR-AL-DR). C57BL/6 male and female mice were used to examine sex differences (N = 10/sex/group). Mice were fed AL or DR from 3 until 15 months (baseline) and each dietary crossover lasted approximately 5 months.

**Results:**

In females, body and fat mass were proportional to the changes in feeding regime and plasma insulin and glucose tolerance were unaffected by the crossovers. However, in male mice, glucose tolerance and plasma insulin levels were reversed within 6 to 12 weeks. When males returned to AL intake following 5 months DR (AL-DR-AL), body mass was maintained below baseline, proportional to changes in fat mass. Glucose tolerance was also significantly better compared to baseline.

**Conclusions:**

Male mice retained a metabolic memory of 5 months of DR feeding in terms of reduced body mass and improved glucose tolerance. This implies that some of the beneficial effects induced by a period of DR in adult life may be beneficial, even when free feeding is resumed at least in males. However, under continuous DR, lifespan extension was more prominent in females than in males.

## Background

In mammals, pancreatic β cells secrete insulin in proportion to the concentration of circulating glucose. Insulin then stimulates glucose uptake into skeletal muscle and adipose tissue and decreases hepatic glucose production. Defects in insulin secretion by the β cells can lead to hyperglycemia and the onset of type 2 diabetes [1]. Chronic dietary restriction (DR) in C57BL/6 inbred mice has been shown to have beneficial effects on glucose tolerance [2,3]. Additionally, improved insulin sensitivity and reductions in plasma insulin during DR have been linked to the life extending effects of DR in mice [4,5].

Although chronic DR is known to lead to improvements in glucose tolerance and insulin sensitivity, perhaps more relevant for humans is whether only a short period of DR has the same effects. Previous data show this is indeed the case with only a short period of DR (*ad libitum* (AL)-DR crossover), in people with type 2 diabetes [6] and in rodents [7-9].

However, very little is known about whether the beneficial effects induced by short-term exposure to DR can have a ‘metabolic memory’ when AL feeding is resumed (AL-DR-AL) and vice versa: whether the effects of long-term DR can be reversed by a period of AL feeding (DR-AL-DR). We performed these crossovers in laboratory mice to determine the effects of such switches in feeding regimes on body composition as well as glucose and insulin sensitivity. The majority of studies show that male rodents are more insulin resistant than females [10,11]. Therefore, we also aimed to determine whether there was sexual dimorphism in glucose tolerance and insulin sensitivity in response to the dietary crossovers.

We show striking sexual dimorphism, whereby glucose tolerance and insulin sensitivity of female mice was relatively unperturbed by the crossovers. However, in males these parameters improved within 6 to 12 weeks of the crossover to DR and vice versa. Improved glucose tolerance and reduced body mass were retained in males after returning to AL feeding following 5 months DR, suggesting that several of the potentially beneficial effects of short period of DR were retained.

## Results

### Body mass and body composition

C57BL/6 mice were randomly assigned to a DR or an AL group at 3 months of age (day 0 of the experiment). The majority of animals remained in their group until they were killed for experiments at predetermined time points or died naturally. In addition, ten mice per group were assigned to a double-crossover experiment with the first crossover at day 365 (15 months of age) and the second (reverse) crossover at day 505 (about 20 months of age). These mice were then killed at 25 months of age.

**Figure 1:**
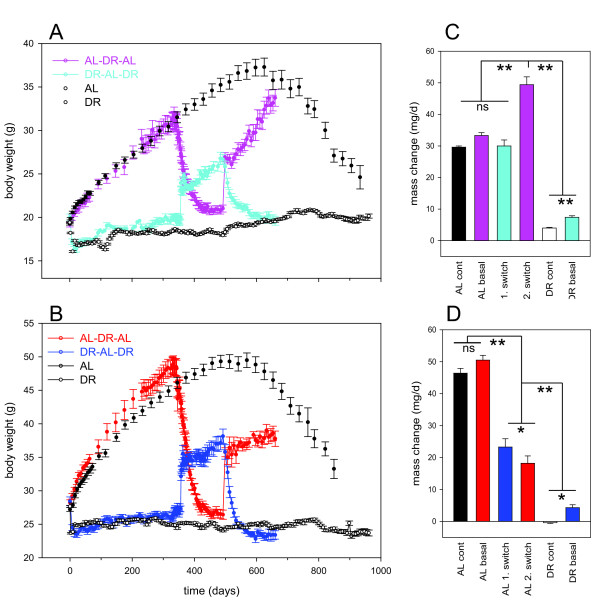
**Body mass changes in double-crossover and long-term control mice. (A,B)** Body mass curves for female (A) and male (B) mice. Prior to the experiment starting (day −7), when mice were 3 months old there was no difference in body mass or food intake between the groups (*P* >0.05). A 40% food restriction was initiated in the dietary restricted group on day 0. Data represent means ± SEM from N = 6 to 10 mice/group in the crossover groups and from 280 (at start) to 48 (at the end) mice in the control groups. **(C,D)** Rates of body mass change with time under the indicated treatments in females (C) and males (D). Rates were calculated by linear regression for time points within an approximately linear range and are means ± SEM. Colors are as in (A) and (B). Asterisks denote significant differences between groups (*P <0.05, **P <0.001 assessed using one-way analysis of variance (ANOVA)/Holm-Sidak).

We first compared body mass trajectories in the crossover groups to large single treatment control cohorts (AL-only or DR-only groups, Figure 1). Food intake in the crossover males under AL was higher than the average AL only group, resulting in higher body mass after 1 year of the experiments (Figure 1B) and on average a higher degree of restriction (around 45%). However, rates of body mass changes before the first crossover were not significantly different between the groups selected for crossover and the control cohorts (Figure 1C,D). The body weights of male AL control mice peaked slightly before that of female AL controls. Weight loss at high age was also seen in DR control mice, however, it was of lower magnitude and its onset was delayed by about 150 to 200 days (Figure 1A,B).

During the crossovers to AL, food intake initially increased (hyperphagia) in both sexes. However, it stabilized in females at baseline level, while it remained higher than baseline in males (*P* <0.001 [see Additional file 1: Figure S1]). Following the first crossover into AL, body mass increased over the 5 months (repeated measures: *P* <0.001) in both sexes. However, despite increased food intake in males after the DR-AL crossover, body mass increased more slowly after DR than in AL-only animals (Figure 1D). This was not the case for females, where body mass gain after switch to AL was at the same rate as in AL-only controls (Figure 1C). This difference between sexes was confirmed after the second crossover to AL feeding: Following 5 months of adult-onset DR, body mass of females increased even faster than in AL-only controls, while rates of increase remained low for males (Figure 1C,D) despite increased food intake [see Additional file 1: Figure S1]. Accordingly, females returned to baseline body mass after 5 months while in males, body mass was maintained below baseline until the end of the experiment (*P* <0.001).

Following the crossovers from AL to DR, males showed stronger responses in body weight than females, approaching the body weight of DR-only animals more closely (following the first crossover) or even losing weight below that level (after the second crossover). This might be due to the above-normal food intake in the male crossover mice during AL periods resulting in more severe dietary restriction.

There were no depot-specific changes in fat mass following the first or second crossover in either sex. All changes in fat masses at any point in the experiment were fully proportional to the respective body mass changes. In long-term controls dissected aged 12, 15 or 24 months, the decreased mass of all the organs in DR mice was entirely on account of the reduced body mass. In males there were no significant differences in relative organ mass per total body mass induced by the crossovers. In females, the relative masses of the kidneys were significantly less in DR mice after the first (*P* = 0.003) and second (*P* = 0.011) crossover, and the liver (*P* = 0.044) only after the second crossover.

In summary, males, but not females, maintained low body mass with slow mass gains after return to AL feeding from either early-onset or late-onset DR, despite increased food intake.

### Glucose tolerance

**Figure 2:**
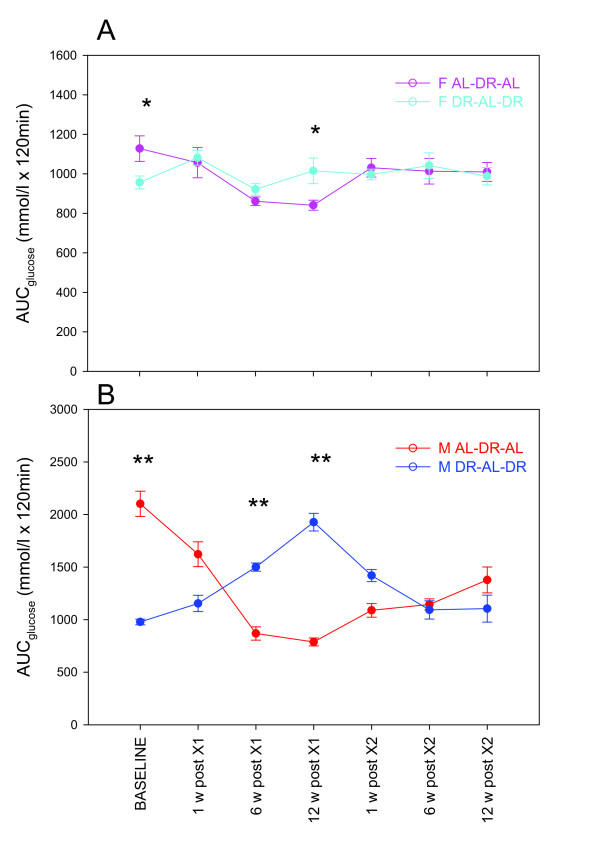
**Glucose tolerance in female (F; (A)) and male (M; (B)) mice during double crossover.** Data were calculated as the area under the curve (AUC) following a glucose injection and represent means ± SEM from N = 6 to 10 mice/group. Asterisks denote significant differences between groups (*P <0.05; **P <0.001), assessed using one-way analysis of variance (ANOVA). In male AL-DR-AL mice, glucose tolerance was significantly better at the end than at baseline (*P* <0.001). AL = *ad libitum*; DR = dietary restriction.

We next assessed glucose tolerance immediately before and at different time points after first and second crossover. DR mice were more glucose tolerant than AL mice at baseline in both sexes (Figure 2). In female mice, glucose tolerance responded only minimally to changes in the feeding regimen. Females in the DR-AL-DR group maintained the same glucose tolerance levels throughout the experiment (*P* = 0.245). However, females that were crossed over to DR at 15 months of age improved their glucose tolerance resulting in a significant difference (*P* = 0.022) by 12 weeks after the first crossover. No differences were detectable between the groups following the second crossover.

Glucose tolerance in male mice was more responsive to changes in the feeding regime and showed highly significant changes over time within both groups (*P* <0.001). Following either the first or second crossover to DR, glucose tolerance improved fully within 6 weeks (*P* <0.001, Figure 2B). However, following the inverse crossover to AL, glucose tolerance in males reached AL baseline levels only at 12 weeks after the first crossover and remained significantly improved over baseline up to the end of the experiment after the second crossover (*P* = 0.001, Figure 2B).

Together, these data show that adult-onset DR induced a lasting improvement in glucose tolerance in males, which paralleled the maintenance of low body mass. Conversely, a short-term reversion from DR did not result in longer lasting impairment in glucose tolerance. Glucose tolerance in females was much less influenced by feeding regime.

### Fasting glucose, insulin and insulin sensitivity

**Figure 3:**
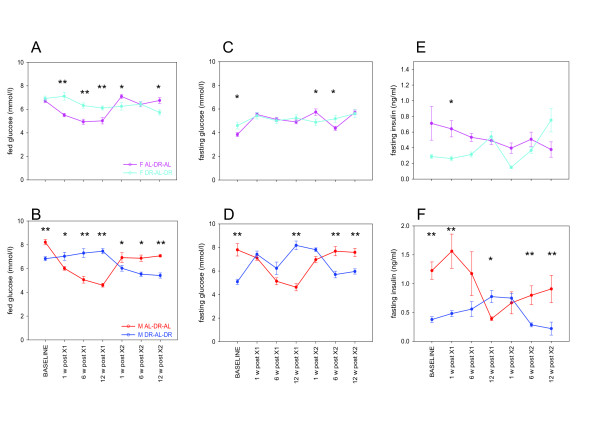
**Glucose and insulin levels in female (F; (A,C,E)) and male (M; (B,D,F)) mice during double crossover.** Fed glucose **(A,B)** was measured at 11.30 am when mice were postprandial. Fasting glucose **(C,D)** and fasting insulin **(E,F)** were measured following an overnight fast. Data represent means ± SEM from N = 6 to 10 mice/group. Asterisks denote significant differences between groups (*P <0.05; **P <0.001), assessed using one-way analysis of variance (ANOVA).

In agreement with the sex differences seen in body mass maintenance and glucose tolerance, males and females also showed different responses in fed and fasting glucose concentrations and insulin levels to dietary change. Male DR mice had lower fed (Figure 3B) and fasting glucose (Figure 3D) and insulin (Figure 3F) levels at baseline (all *P* <0.001) and all parameters were highly affected by the crossovers (*P* <0.001). With the exception of fasting insulin after the first crossover, all parameters were completely reverted from baseline AL levels within 6 weeks after the crossover to DR. When male mice were crossed back to AL from either long-term or short-term DR, changes in glucose were also completed within 6 weeks after the crossover. However, fasting insulin levels in male mice crossed over to AL after a period of DR remained below the AL baseline for at least 12 weeks, both after long-term and short-term DR. Insulin sensitivity as evaluated by the homeostasis model assessment 2 (HOMA2) protocol was reversed by 12 weeks (*P* <0.001) after the first cross and by 6 weeks (*P* = 0.008) after the second crossover in males. By the end of the experiment, males exposed to a (DR-AL-DR) regime had significant improvements in insulin sensitivity compared to baseline (*P* = 0.001) [see Additional file 2: Figure S2]. In males only, there was a significant positive correlation between fasting insulin and glucose (AL-DR-AL: R^2^ = 0.075, *P* = 0.050; DR-AL-DR: R^2^ = 0.177, *P* = 0.001) and between AUC of glucose clearance and insulin concentrations (AL-DR-AL: R^2^ = 0.174, *P* = 0.001; DR-AL-DR: R^2^ = 0.179, *P* = 0.003).

In female mice, fed glucose levels were reduced following the first crossover to a DR regime and this was reverted by crossing back to AL (Figure 3A). Fasting glucose (Figure 3C) and insulin (Figure 3E, borderline significance) were lower at baseline as expected. However, changes over time and differences between the groups in fasting glucose and insulin concentrations were generally too small in females to show consistent patterns with the available numbers of animals.

In male mice, glucose tolerance and insulin sensitivity were strongly affected by the crossover regimes. To establish the impact of body mass on these effects, correlations between body mass and the measured parameters were calculated. Body mass was positively related to fasting glucose and insulin (glucose; AL-DR-AL: R^2^ = 0.355, *P* <0.001; DR-AL-DR: R^2^ = 0.380, *P* <0.001 and insulin; AL-DR-AL: R^2^ = 0.186, *P* = 0.002; DR-AL-DR: R^2^ = 0.270, *P* <0.001). There was therefore a significant negative correlation between body mass and insulin sensitivity (AL-DR-AL: R^2^ = 0.343, *P* <0.001; DR-AL-DR: R^2^ = 0.351, *P* <0.001). There was a significant positive correlation between AUC of glucose clearance and body mass in males (AL-DR-AL: R^2^ = 0.603, *P* <0.001; DR-AL-DR: R^2^ = 0.213, *P* <0.001). This was also significantly correlated in females, but only in the AL-DR-AL group (R^2^ = 0.356, *P* <0.001) [see Additional file 3: Figure S3].

Together, these data show that even a short period of DR induces improvements of fasting insulin levels, glucose tolerance and body mass maintenance that can last considerably in males while they are of smaller magnitude and more quickly reversed in females.

### DR effects on longevity and tumor prevalence

**Figure 4:**
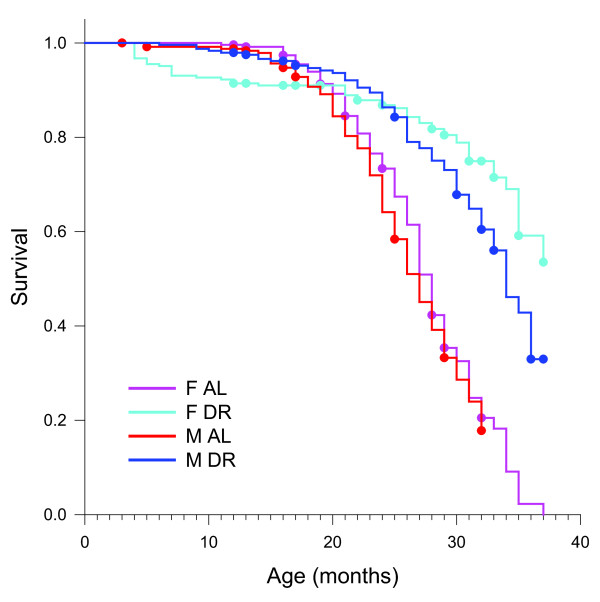
**Lifespan in*ad libitum*(AL) only and dietary restriction (DR) only male and female control mice.** Right - censored Kaplan-Meier curves, dots represent censoring events.

Lowering of circulating insulin levels and improvement of glucose tolerance are seen as important mediators of the lifespan-improving and health-improving effects of DR [4,5]. Given the sexual dimorphism in the response of these parameters to DR shown above, different degrees of health-related and lifespan-related effects of DR between males and females might be expected. The present study was not designed to analyze long-term health and lifespan effects after short-term DR, and frequencies of death occurring in the four crossover groups until the end of the experiment were not significantly different (data not shown). However, data on lifespan (Figure 4) and tumor prevalence at death (Table 1) are available from the large AL and DR only control cohorts. Lifespans of male and female mice under AL feeding were not different from each other (*P* = 0.192). Median lifespans were 27 ± 0.61 months for AL males and 28 ± 0.41 months for AL females. DR improved survival in both sexes, but the extension was significantly greater in females (*P* = 0.0163). Median lifespan increased by about 26% to 34 ± 0.78 months in males, and by at least 32% to >37 months in females. Under AL feeding, tumor prevalence increased sharply in both sexes after 17 months of age, but percentages of tumor-bearing mice remained lower in males than in females over their whole remaining lifespan (Table 1). DR strongly reduced tumor prevalence in females. In males, however, DR appeared to postpone tumor incidence but did not reduce the percentages of mice bearing neoplasms after 20 months of age (Table 1).

**Table 1:** Number (%) of neoplasm-bearing mice/number of mice examined

**Age at death**	**F AL**	**F DR**	**M AL**	**M DR**
12 ± 1 months	2/22 (9.1)	2/19 (10.5)	0/19 (0.0)	0/24 (0.0)
17 ± 1 months	8/41 (19.5)	0/15 (0.0)	4/45 (8.9)	0/34 (0.0)
20 ± 1 months	13/21 (61.9)	0/16 (0.0)	9/20 (45.0)	1/5 (20.0)
23 ± 1 months	24/47 (51.1)	7/35 (20.0)	12/31 (38.7)	5/11 (45.5)
26 ± 1 months	18/34 (52.9)	0/6 (0.0)	15/61 (24.6)	13/48 (27.1)
29 ± 1 months	16/30 (53.3)	3/31 (9.7)	2/24 (8.3)	4/25 (16.0)
32 ± 1 months	22/37 (59.5)	15/41 (36.6)	11/36 (30.6)	8/58 (13.8)
35 ± 1 months	1/8 (12.5)	5/36 (13.9)		7/34 (20.6)
37 months		2/20 (10.0)		

## Discussion and Conclusion

This study addresses two related questions: firstly, is there a ‘metabolic memory’ if mice are switched between AL and DR feeding regimens?

Work in flies and rats has shown that the switch between DR and AL feeding regimes can be a very dynamic process, particularly in terms of survival and metabolic status with reversal of feeding regimes resulting in a rapid change in ageing trajectory in *Drosophila*[12] and in rats [13]. Markers of oxidative damage were found to be reversed with the feeding regime in flies [14], and in the brains of mice [15]. In mice, gene expressions profiles in liver, muscle and hypothalamus shifted quickly in correspondence to a new feeding regime [16], including genes involved in metabolism and growth control. In rats, effects of short-term to medium-term early-onset DR were found to be obliterated by a later period of AL feeding [13].

We found that male mice retained a ‘metabolic memory’, that is, improved body mass maintenance, glucose tolerance and fasting insulin levels for up to 5 months after a period of adult-onset DR. A similar experiment has been performed in the same strain of male mice whereby AL mice were crossed to DR feeding at 11 months of age and vice versa [17]. No second crossover was performed in this study, but follow-up time was for 10 months after crossover. This study also shows that in males crossed from DR to AL, body mass remained below long-term AL levels. Fat mass remained below control levels for at least 6 months after crossover to AL. Importantly, glucose tolerance did remain significantly improved compared to long-term AL controls for the whole observation period (10 months after the crossover). This reinforces the suggestion that metabolic memory of DR is retained in male mice in terms of improved glucose tolerance.

The second question was, are sex-specific responses in insulin, glucose tolerance and body weight maintenance associated with the longevity effect of DR?

In our study, AL females showed better glucose tolerance, fasting glucose and fasting insulin levels than AL males, and these were largely unaffected following short-term or long-term DR. In contrast, glucose and insulin levels and glucose tolerance were more responsive to periods of DR in males confirming published data [9] and together with body mass maintenance, showed lasting improvements following a period of DR. Fasting glucose and insulin concentrations positively correlated with body mass, as previously reported [18], with a subsequent negative correlation between body mass and insulin sensitivity [7]. This suggests that the function of pancreatic β cells to secrete insulin was not impaired in AL mice.

There is extensive evidence showing general sexual dimorphism in insulin sensitivity. Several factors could be responsible for this. One is the influence of sex hormones; testosterone has a direct effect upon pancreatic islet function by favoring insulin gene expression and insulin release [19]. Estrogen has beneficial effects on glucose tolerance and insulin resistance: for example, women are more likely to develop diabetes after menopause but hormone replacement therapy can ameliorate this tendency [20], and in ovariectomized mice, administration of estrogen protected against glucose intolerance induced by high fat diet in an estrogen receptor alpha dependent manner [21]. Furthermore, sexual dimorphism in adipokines that regulate insulin sensitivity such as resistin, leptin, adiponectin, retinol binding protein 4 (RBP4) and glucocorticoids has been shown [22-26]. Additional factors, such as the capacity of peripheral organs, primarily skeletal muscle, to uptake glucose might also influence gender differences in insulin sensitivity and glucose tolerance.

There is also ample evidence for a sexual dimorphism in response to lifespan-extending manipulations. In mice, the effects of lifespan extension by deletion of insulin substrate receptor I [27], or feeding with the mammalian target of rapamycin (mTOR) inhibitor rapamycin [28] were found to be more robust in females than males. Similarly, reduction of the activity of the insulin-signaling pathway [29], or the mTOR pathway by deletion of S6K [30] extended lifespan only in female but not in male mice. Also in *Drosophila*, females tend to show enhanced responses to various lifespan extension manipulations as compared to their male counterparts. For example, dFOXO overexpression in fat body extended lifespan in females but not in males in *Drosophila*[31]. Female flies show greater extension of lifespan by DR than males [32]; the reason is not completely clear while the reduction in egg laying activity in female DR flies has been postulated to be one possible explanation. There is contradictory evidence regarding a sexual dimorphism in the lifespan response to DR in C57BL/6 mice. Blackwell [33] reported identical lifespan between the sexes in both AL control and DR mice. Using single-housed animals, Turturro *et al*. [34,35] showed larger lifespan extension under DR in females, however, this was driven by a shorter lifespan in AL females as compared to AL males. Group-housed animals in our cohort reached higher median ages already under AL, which were not different between sexes. However, lifespan was more extended by DR in females than in males.

It had been suggested that the effects of DR on lifespan are mitigated through circulating insulin levels, which may reduce insulin signaling [4,5]. However, longer lifespan and better glucose tolerance are not always associated with each other. For example, insulin receptor substrate 1 null mice had extended lifespan together with lifelong mild glucose intolerance [27], and Harper *et al*. [36] reported that a long-lived mouse stock had impaired glucose tolerance compared to control mice. According to our data on AL females and males, the lower fasting insulin levels in AL females are not associated with longer lifespans, and tumors are, if anything, more frequent in AL females relative to AL males. In case of DR mice, lifelong DR results in greater extension of lifespan and more prominent tumor suppression in females than in males despite both sexes displaying indistinguishable glucose tolerance and insulin sensitivity. The interconnections between sexual dimorphisms in metabolic and lifespan regulation appear to be more complex than originally thought. Whether the retention of a ‘metabolic memory’ in male mice after a brief period of DR in mid life improves their health/lifespan beyond the period in which they maintain better glucose tolerance and lower body mass remains to be elucidated.

## Methods

### Mice

All mice were inbred C57BL/6 (Harlan, Blackthorn UK) and both males and females were used. Ethical approval was granted by the LERC Newcastle University, UK. The work was licensed by the UK Home Office (PPL 60/3864) and complied with the guiding principles for the care and use of laboratory animals.

Mice were housed in same-sex cages in groups of 4 to 6 (56 × 38 × 18 cm, North Kent Plastics, Kent, UK) and individually identified by an ear notch. They were provided with sawdust, paper bedding and environmental enrichment (a plastic house). Mice were housed at 20 ± 2°C under a 12 h light/12 h dark photoperiod with lights on at 7.00 am. The diet used was standard rodent pelleted chow (CRM (P); Special Diets Services, Witham, UK) for AL-fed mice and the same diet, but as smaller pellets were offered to DR mice. The smaller pellet size reduced competition for food. DR mice were offered 60% of AL intake (calculated based on average food intake in 90 control AL mice between 5 and 12 months of age) as one ration at 9.30 am daily. All mice were fed AL until 3 months of age and then split into AL or DR groups, matched for body mass and food intake (N = 10/sex/group for crossover groups). At 15 months of age, mice were crossed over from DR to AL or AL to DR. After a further 140 days (about 20 months of age), these mice were returned to the original feeding regime for a further 160 days, until they were killed at an age of 25 months, resulting in four experimental groups: male AL-DR-AL, male DR-AL-DR, female AL-DR-AL and female DR-AL-DR. During the experiment three females and four males died or were killed from the AL-DR-AL group, and one female and four males from the DR-AL-DR group. Effects of DR on body mass, survival and tumor prevalence were monitored in long-term controls which were fed only DR or AL from 3 months of age, comprising 280 mice/sex/group in total. These were either killed at predetermined ages or left to die naturally. All mice were dissected and macroscopically examined for tumor prevalence at death.

### Body mass, body composition and food intake

Body mass and food intake were measured at least once a month in AL mice and once a week in DR mice (± 0.01 g; Sartorius top-pan balance, Epsom, UK). Mean food intake of each AL cage was measured by weighing the contents of the food hopper on 2 consecutive days and this amount divided by the number of mice in the cage. Food intake in the double-crossover mice over the course of the experiment is shown in Additional file 1. Average food intake in the crossover males under AL was higher than in the AL only controls. However, during the last weeks before crossover, the degree of DR was not significantly different from 40% in either males or females. Full body dissection was performed at all endpoints and the organs weighed (Ohaus analytical balance, ± 0.0001 g; Ohaus Corp., NJ, USA): brain, heart, lungs, thymus, quadriceps, tail, caecum, liver, kidneys, spleen, gonads and pancreas. Also, large intestine and small intestine mass (after flushing with saline) and length were determined. To assess fat deposition, six fat depots were also fully dissected and weighed: retroperitoneal, gonadal, mesenteric, subcutaneous, subscapular and brown adipose tissue (BAT).

### Glucose tolerance test

A glucose tolerance test (GTT) was performed on each individual in the crossover experiment at 15 months old (baseline) and then at 1, 3 and 12 weeks after the first crossover and 1, 3 and 12 weeks after the second crossover. The GTT was performed on fasting mice by removing all food from AL mice at 6.00 pm the evening before (15.5 h fasting) and withholding the daily food ration from DR mice until after the test. Drinking water was available throughout. A 20% glucose solution was prepared fresh each morning using d-glucose (G-5767, Sigma-Aldrich, St Louis, MO, USA) and sterile filtered water. A fasting blood sample was collected by placing each mouse in a restrainer and nicking the tail vein with a scalpel blade. A total of 200 μl of blood was collected from each animal in a microvette container lined with lithium-heparin (Microvette, Sarstedt AB, Landskrona, Sweden). Blood was centrifuged and the resultant plasma stored at −80 °C. The fasting blood glucose level (mol/l) (time-point 0) was determined using a Glucometer (ACCU-CHEK Aviva Nano, Mannheim, Germany) from a further approximately 2 μl of blood. Then, mice were injected intraperitoneally with 2 g/kg body mass of the glucose solution. At 15, 30, 60 and 120 minutes post injection the blood glucose level was measured using blood from the tail vein on the Glucometer as above. At the end of the GTT, DR mice were fed their daily ration and food was replenished in AL food hoppers. Glucose tolerance was expressed as the area under the curve over the 120-minute test duration. On a separate occasion, at least 3 days before or after a GTT, fed glucose blood concentrations were measured using a drop of blood from the tail on the Glucometer as above, at 11.30 am to ensure mice were postprandial.

### Fasting plasma insulin levels and insulin sensitivity

Using the fasting plasma collected prior to the GTT, insulin concentrations were measured using an ultrasensitive mouse insulin ELISA kit (CrystalChem Inc., Downers Grove, IL, USA). All samples were run in duplicate and a number of additional standards were included because the concentrations measured were close to the detection limit. Insulin sensitivity was estimated using the updated homeostatic model assessment (HOMA2) model which gives an estimate of insulin sensitivity using fasting plasma insulin and glucose concentrations [37]. This model can be used as a comparison between experimental groups as a measure of insulin sensitivity in rodents [38].

### Statistical analysis

All statistical analyses were performed using Minitab V. 16 (Minitab Inc., State College, PA, USA) and Sigmaplot V. 11.0 (SPSS, Chicago, IL, USA). Repeated measures analysis of variance (ANOVA) was used when analyzing changes in body mass and food intake data over time. Fat and organ mass co-vary with body mass, therefore mass was used as a covariate in a general linear model (GLM) to control for these effects. One-way ANOVA was used to find differences between groups. A Tukey comparison was included in the one-way ANOVA to determine differences between all the measured timepoints within the same group. Linear least squares regression was used to find significant correlations between two continuous factors. Kaplan-Meier survival curves were compared by log-rank test. Differences were considered significant when *P* ≤0.05.

### Availability of supporting data

## Competing interests

The authors declare they have no competing interests.

## Authors’ contributions

KMC participated in the design of the study, performed the experimental work, carried out data analysis and interpretation and wrote the manuscript. SM and TVZ participated in the design of the study, data analysis and interpretation and wrote the manuscript. CW contributed to the experimental work. All authors read and approved the final manuscript.

## Supplementary Material

Additional file 1 **Food intake of female (F; (A)) and male (M; (B)) mice in the double crossover groups.** Data are mean ± SD from 6 to 10 mice /group. Click here for file

Additional file 2 **Insulin sensitivity calculated using the homeostasis model assessment 2 (HOMA2) model in female (F; (A)) and male (M; (B)) mice.** Data were calculated using values of fasting glucose and insulin concentrations. In females, the phenotype was never completely reversed. In males, there was complete reversal of the phenotype by 12 weeks after the first cross, and 6 weeks after the second, whereby dietary restriction (DR) mice were significantly more insulin sensitive. Data represent means ± SEM from N = 6 to 10 mice/group. Asterisks denote significant differences between groups (*P <0.05; **P <0.001), assessed using one-way analysis of variance (ANOVA). Click here for file

Additional file 3 **The relationship between glucose tolerance measured as area under the curve (AUC) of glucose clearance and body mass in female (F; (A)) and male (M; (B)) mice.** Glucose tolerance was highly dependent on body mass, particularly in males, whereby heavier mice were less glucose tolerant. Data collected at each timepoint were combined to give N = 50 to 70 data points/group. Correlations were assessed using linear regression, and deemed significant when *P* <0.05. Click here for file
