# Supplementary figures and images for: Male mice retain a metabolic memory of improved glucose tolerance induced during adult onset, short-term dietary restriction
Source: Longev Healthspan. 2012 Sep 3;1:3. doi: 10.1186/2046-2395-1-3 (PMC3886267; doi:10.1186/2046-2395-1-3)

## Slide 1
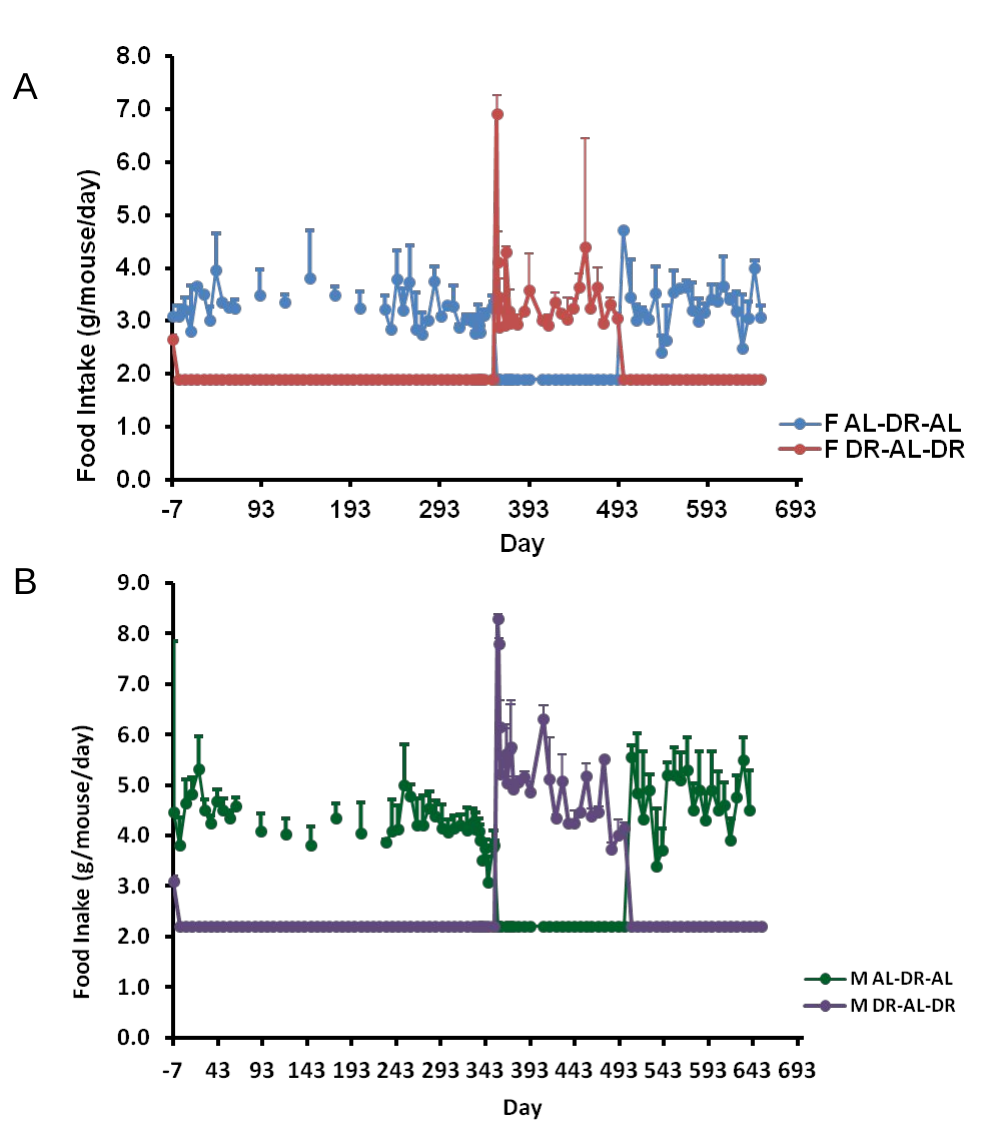

A
B

Supplement: Additional file 1 — Food intake of female (F; (A)) and male (M; (B)) mice in the double crossover groups. Data are mean ± SD from 6 to 10 mice /group. [file 2046-2395-1-3-S1.ppt]

## Slide 1
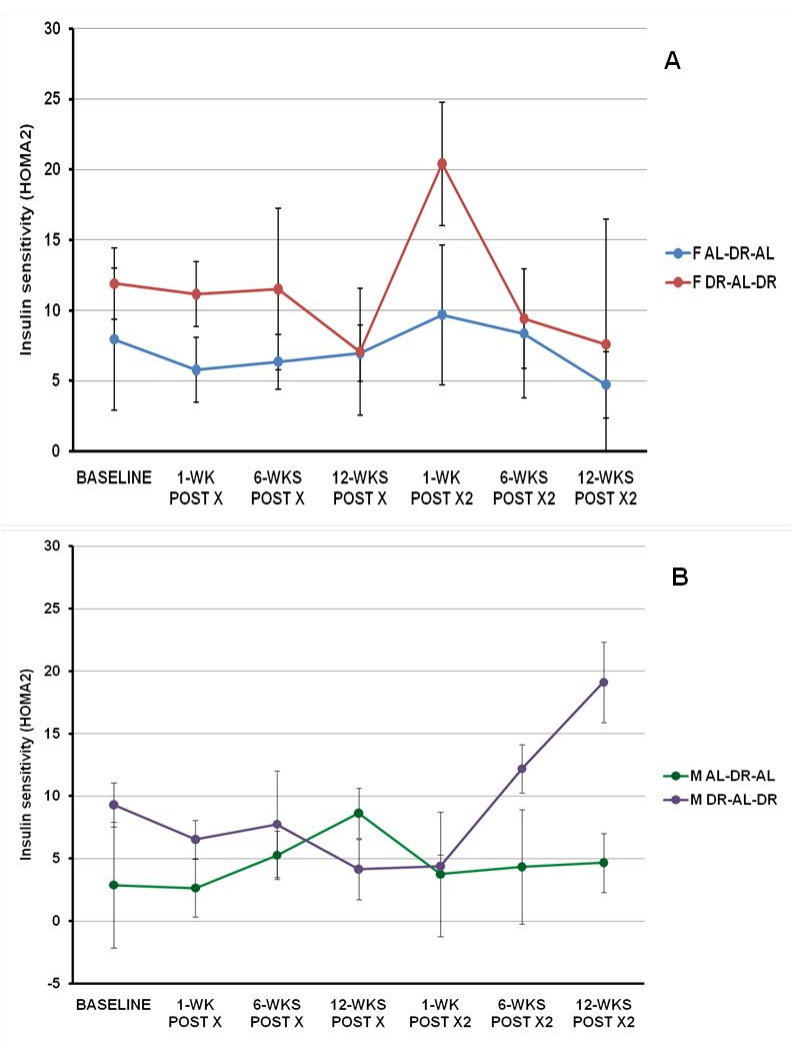

Supplement: Additional file 2 — Insulin sensitivity calculated using the homeostasis model assessment 2 (HOMA2) model in female (F; (A)) and male (M; (B)) mice. Data were calculated using values of fasting glucose and insulin concentrations. In females, the phenotype was never completely reversed. In males, there was complete reversal of the phenotype by 12 weeks after the first cross, and 6 weeks after the second, whereby dietary restriction (DR) mice were significantly more insulin sensitive. Data represent means ± SEM from N = 6 to 10 mice/group. Asterisks denote significant differences between groups (*P <0.05; **P <0.001), assessed using one-way analysis of variance (ANOVA). [file 2046-2395-1-3-S2.ppt]

## Slide 1
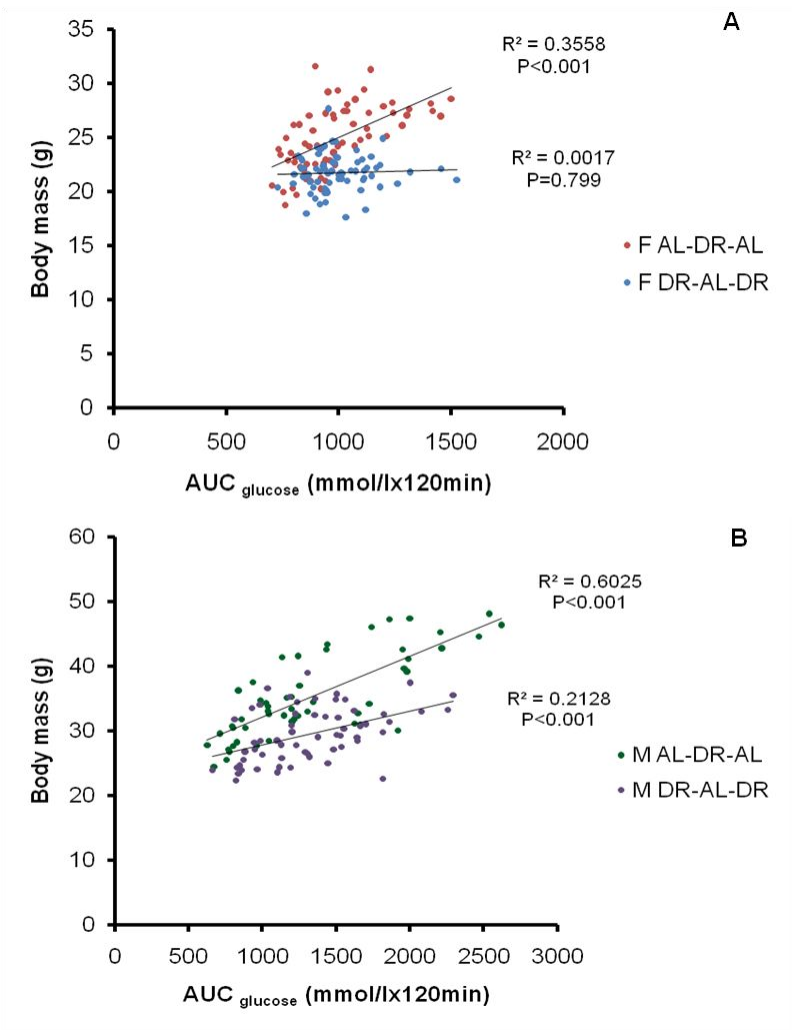

Supplement: Additional file 3 — The relationship between glucose tolerance measured as area under the curve (AUC) of glucose clearance and body mass in female (F; (A)) and male (M; (B)) mice. Glucose tolerance was highly dependent on body mass, particularly in males, whereby heavier mice were less glucose tolerant. Data collected at each timepoint were combined to give N = 50 to 70 data points/group. Correlations were assessed using linear regression, and deemed significant when P <0.05. [file 2046-2395-1-3-S3.ppt]
